# Molybdenum-Doped ZnO Thin Films Obtained by Spray Pyrolysis

**DOI:** 10.3390/ma17092164

**Published:** 2024-05-06

**Authors:** Pavlina Bancheva-Koleva, Veselin Zhelev, Plamen Petkov, Tamara Petkova

**Affiliations:** 1 Physics Department, University of Chemical Technology and Metallurgy, 8 Kliment Ohridski Blvd., 1756 Sofia, Bulgaria; p.petkov@uctm.edu; 2 Institute of Electrochemistry and Energy Systems, Bulgarian Academy of Sciences, Akad. G. Bonchev Str. Bl. 10, 1000 Sofia, Bulgaria; v.zhelev@iees.bas.bg (V.Z.); tpetkova@iees.bas.bg (T.P.)

**Keywords:** Mo-doped ZnO, thin film, spray pyrolysis

## Abstract

A batch of ZnO thin films, pure and doped with molybdenum (up to 2 mol %), were prepared using the spray pyrolysis technique on glass and silicon substrates. The effect of molybdenum concentration on the morphology, structure and optical properties of the films was investigated. X-ray diffraction (XRD) results show a wurtzite polycrystalline crystal structure. The average crystallite size increases from 30 to 80 nm with increasing molybdenum content. Scanning electron microscopy (SEM) images demonstrate a smooth and homogeneous surface with densely spaced nanocrystalline grains. The number of nuclei increases, growing over the entire surface of the substrate with uniform grains, when the molybdenum concentration is increased to 2 mol %. The estimated root mean square (RMS) roughness values for the undoped and doped with 1 mol % and 2 mol % of ZnO thin films, defined by atomic force microscopy (AFM), are 6.12, 23.54 and 23.83 nm, respectively. The increase in Mo concentration contributes to the increase in film transmittance.

## 1. Introduction

In recent times, conductive and transparent ZnO films have gained more attention due to their properties, such as light-emitting semiconductivity, relatively low deposition temperature, as well as having a high value of exciton binding energy, etc. [1]. These properties led to the films having found many applications including acoustic sensors, high temperature thermoelectric devices, active emitters in LEDs and laser diodes, TFT for the real-time sensing of gas molecules, etc. [2,3].

Different grow techniques, such as sol-gel, thermal evaporation, chemical vapor deposition (CVD), ultrasonic spray pyrolysis (USP) and magnetron sputtering, could be used to grow thin ZnO films [4,5,6,7,8,9]. The spray pyrolysis technique is attractive because it is cost-effective, with an easy experimental setup, and it allows wide-area deposition, etc. [4,10]. Zinc oxide is an n-type semiconductor with a wide band gap, high free exciton binding energy (60 meV), wide range of resistivity values and high transparency in the visible region [1]. Because of their optical properties, these films are suitable for use as transparent conductive films when they are doped with a proper dopant, reducing the band gap. One of the metal oxide semiconductors is ZnO, whose application in the field of optoelectronics is quite popular [11]. Nowadays, the usage of position-sensitive photo-detectors has attracted great interest in many areas. These instruments are applicable in devices for the precise measurement of linear displacements, vibration indication, devices for the monitoring of light-emitting objects, devices for determining roughness, etc. Their structure is “semiconductor—thin dielectric—transparent conductor”, such as the “Si–SiO_2_–metal oxide” structure [12].

To date, relatively little attention has been directed towards investigating pure and Mo-doped ZnO thin films compared to SnO_2_ thin films, which were obtained by spray pyrolysis [13,14]. Molybdenum (Mo) is a dopant with the ability to enhance the conductivity and transparency of zinc oxide thin films [15]. With a smaller radius (0.062 nm) when compared to Zn (0.083 nm), Mo is highly suitable for doping into the ZnO matrix. It has the ability to donate four electrons to the free carriers owing to the significant valence difference between Mo^6+^ ions and substituted Zn^2+^ ions, thereby providing sufficient free carriers and influencing the ion scattering effect [1]. What is more, Mo exhibits multiple valence states, and that allows the contribution of multiple carriers by a single Mo dopant atom [16]. The influence of Mo concentration on the physical properties of ZnO thin films was studied by Swapna and Kumar [17]. Our work aims to investigate the growth of thin films using different precursors with a technology we have developed.

## 2. Experimental

Glass and silicon substrates were used for the deposition of undoped and Mo-doped zinc oxide thin films via the spray pyrolysis technique. MoO_3_ (Alfa Aesar) and zinc acetate dihydrate (Zn(CH_3_COO)_2_.2H_2_O, 99%, Alfa Aesar) were used as precursors with a volume ratio of 1:4 ethanol and distilled water as a solvent. The solution was stirred for 30 min during which a few drops of acetic acid were added for the prevention of precipitation of zinc hydroxide. To achieve the full solubility of MoO_3_, a few drops of HCl and NH_3_ were added and the solution was stirred for another 2 h to obtain a homogenous solution. The solution was maintained at a total concentration of 0.1 M and the concentrations of Mo were 1 mol% and 2 mol%. 

Before the deposition process started, we cleaned the glass substrates with isopropanol and double-distilled water, while silicon substrates underwent cleaning with HF. Deposition of the thin films was conducted using a spray pyrolysis unit developed by V. Zhelev and P. Petkov, patented under N 4384 U1. Argon served as a carrier gas at a pressure of 1.2 bar. Identical experimental conditions were maintained for undoped and Mo-doped films to investigate the effects of the dopant on structural, morphological, and optical properties.

The spray pyrolysis unit’s software enabled the adjustment of various process parameters such as target temperature, nozzle amplitude, distance between nozzle and substrate, nozzle speed, and pressure to optimize the deposition process and ensure homogeneous thin films. Optimal values were determined for the distance between the nozzle and substrate (20–22 cm), spray rate (5 mL/min), nozzle amplitude (45 degrees) and deposition temperature 380 °C for both undoped and doped films.

Structural characterization was performed using an X-ray diffractometer, model Philips APD-15, with data collected at ambient temperature within the range of 2θ = 20–85° using Cu-Kα radiation (λ = 1.54178 Å). Surface morphology was investigated via scanning electron microscopy (SEM) using a Zeiss Evo 10 operating in secondary electrons mode with an accelerating voltage of 25 keV. Chemical composition analysis was conducted using an energy dispersive spectroscopy (EDS) detector Zeiss Smart EDX. Atomic force microscopy (AFM) was employed with an MFP-3D instrument from Asylum Research (Oxford Instruments) to analyze surface morphology and roughness. Optical properties of the thin films were measured at room temperature using a Shimadzu UV-Vis Spectrophotometer Shimadzu UV-1900i.

In order to measure the thickness of the films, we utilized the Zeta-20 3D Optical profiler. This instrument combines a fully integrated microscope with advanced metrology capabilities, enabling precise 3D imaging. Additionally, we employed the thin film analyzer F20 to aid in determining the thickness accurately. To prepare the samples, we etched the films sprayed onto glass substrates using a solution of HCl with distilled water. This method allowed us to obtain reliable measurements of the film’s thickness.

## 3. Results and Discussion

### 3.1. Structural Studies

**Figure 1:**
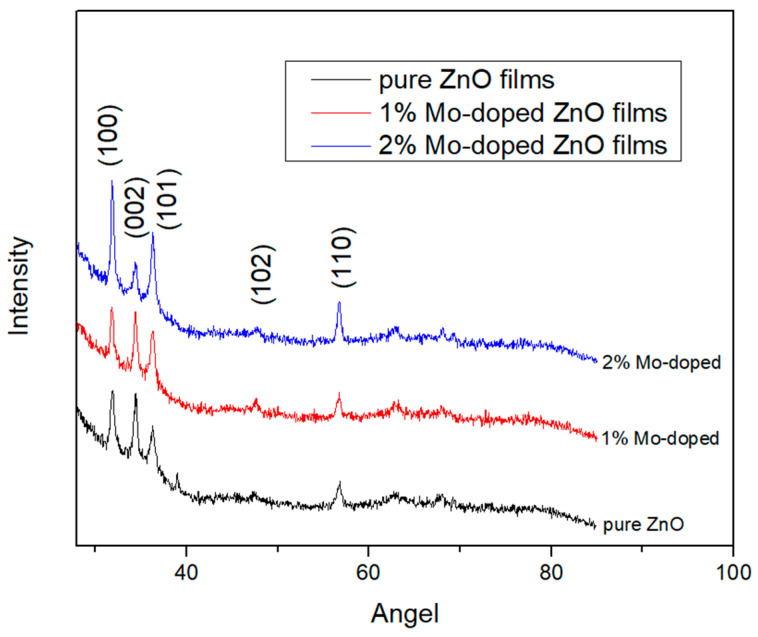
X-ray diffraction patterns of pure and 1% and 2% Mo-doped ZnO thin films.

The XRD measurements indicate that all the films possess a polycrystalline structure with a wurtzite phase and a preferred orientation of the c-axis perpendicular to the substrates (refer to Figure 1). Four distinct peaks corresponding to the (100), (002), (101), and (110) diffraction planes of ZnO are clearly noticeable. No peaks attributable to molybdenum were detected in the XRD patterns, confirming the absence of additional phases in the Mo-doped ZnO thin films.

Upon the addition of molybdenum, the intensities of the (100) and (101) peaks exhibited an increase, while the intensity of the (002) peak revealed a decrease. This trend suggests that the substitution of Zn atoms with isovalent Mo atoms probably induces cell deformation, resulting in changes in the film’s crystallographic properties. It is visible that the (100) plane is preferrable for the growth of an undoped Zn film, while this shows a lack of growth in the (102) and (110) planes, which can be influenced by the substrate. When doping Mo, the planes (100) and (101) are preferred, which corresponds to the results achieved by D. Zhao and et al. [18]. We monitored the decrease in the intensity of the (002) plane with the increase in the Mo-concentration, which might be explained by the fact that the surface energy of the (002) plane is the lowest in the ZnO crystal.

### 3.2. Optical Studies

**Figure 2:**
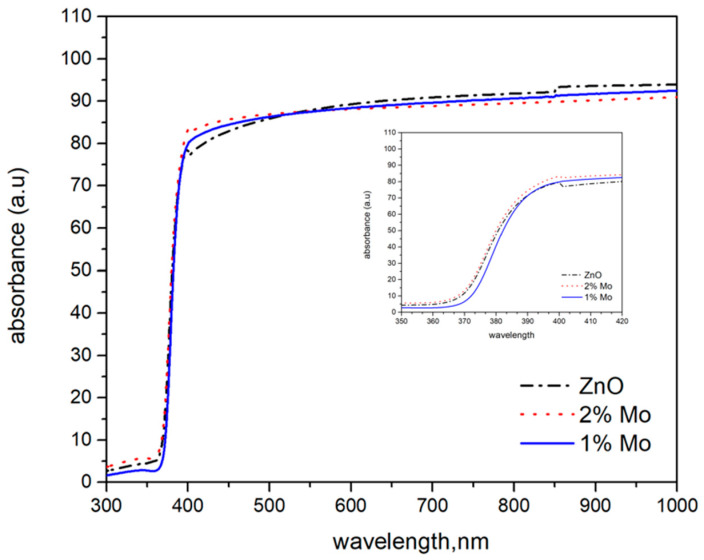
Transmittance spectra of pure and 1% and 2% Mo-doped ZnO thin films.

The transmission and reflectance spectra of pure and Mo-doped ZnO thin films were measured in the range 300–1000 nm. The films of pure ZnO exhibited the lowest optical transmittance in the visible region as compared to the doped films (Figure 2). The addition of molybdenum to ZnO films improves the transparency of the oxide thin films. In principle, when an additive is introduced into the oxide, its transparency is expected to decrease. The effect we observed may be due to the isovalent substitution of zinc with molybdenum in the wurtzite structure of zinc oxide. The layers absorb in the UV region and begin to transmit at the beginning of the visible spectrum around 350 nm. The onset of transmission, also known as the optical edge, changes with the addition of molybdenum. The change is towards longer wavelengths with the addition of 1% molybdenum and a subsequent shift towards lower wavelengths as the molybdenum content increases to 2%. This shift affects the width of the forbidden zone, the values of which show the same trend. Data on film thickness taken from the optical profilometer measurement are presented in Table 1.

Additionally, using the formula

d = λ_1_ − λ_2_/2*n*(λ_1_ − λ_2_), (1)

where *n* is refractive index, and λ_1_ and λ_2_ are the maximal and minimal wavelengths in the visible spectrum, the thickness of the films was calculated, and the data are presented in Table 1.

It is obvious that the data on the measured films’ thickness correspond to the data on the calculated films’ thickness. In regard to the transmission and reflection spectrophotometric data, the refractive index and extinction coefficient of ZnO- and Mo-doped ZnO thin films are estimated in a broad spectral range (300–800 nm). In order to solve a set of two nonlinear equations with two unknowns, which are the real and imaginary parts of the refractive index, we use a derivative approach on a wavelength-by-wavelength basis. The numerical procedure is based on the so-called “trust-region-dogleg” algorithm that is specifically designed as a way to solve nonlinear equations and thus to calculate the optical constants of the film as a function of the wavelength [19,20]. 

To determine the absorption coefficient, we use the relation [21]:

T = (1 − R) exp(−αd),(2)

where T is the transmittance, R is the reflectance, and d is the film thickness. The optical band gap is calculated by applying the Tauc model procedure, with an accuracy ±0.002 eV. [22]:

αhν = B(hν − E_g_)^*n*^ (3)

where the incident photon energy is hν and the optical band gap is E_g_, B is the constant and *n* can be ½, 3/2, 2 or 3. The mode of the interband transition, for example direct allowed, direct forbidden, indirect allowed and indirect forbidden transition, influences the *n* value. To calculate the band gaps of the films, we have used Tauc’s plot by plotting αhν = f(hν) and by extrapolating the linear part of the absorption edge [1]. The result shows that the Eg value decreases with the introduction of the dopant, which could be due to the new energy states between the valence and conduction bands. Additionally, the presence of any defects could be accepted as recombination centers. They form new levels in the band gap, thus lowering it [23]. The slight increase in the band gap value with the increase in the dopant concentration originates from the Burstein–Moss effect. G. Chen et al. observed similar behaviors when magnetron co-sputtering Mo-doped ZnO thin films [16].

### 3.3. Morphological Studies

**Figure 3:**
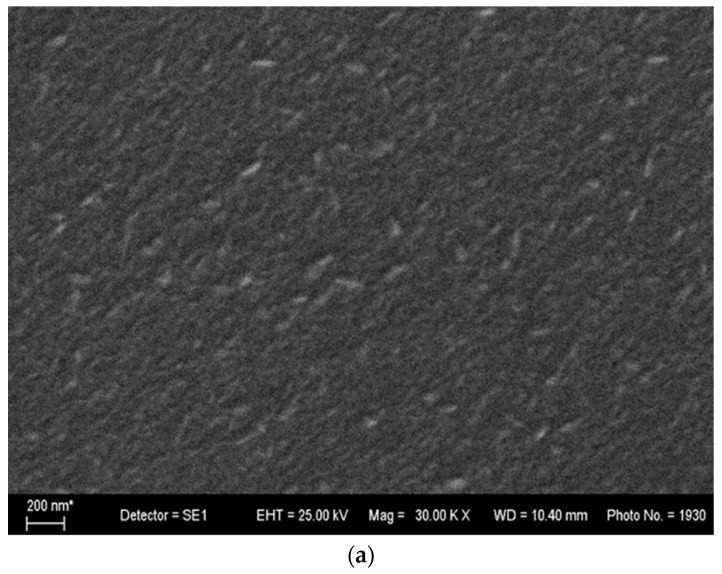

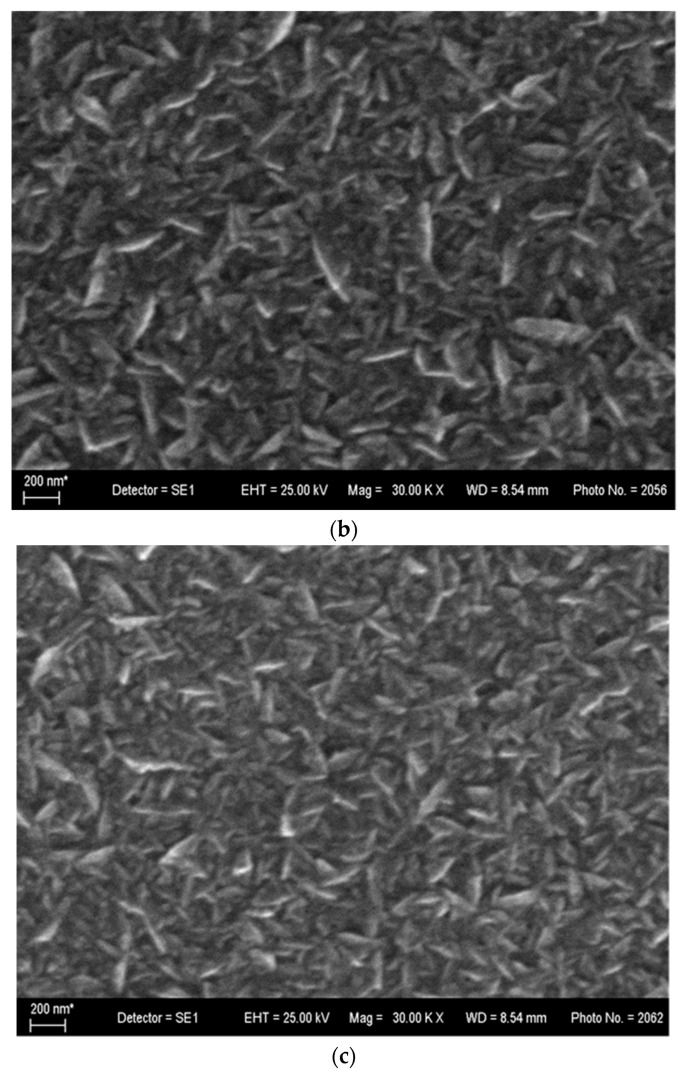
SEM images of (**a**) pure ZnO, (**b**) 2% Mo-doped ZnO thin film and (**c**) 1% Mo-doped ZnO thin film.

The surface morphologies of the undoped and Mo-doped ZnO thin films investigated using scanning electron microscopy (SEM) are presented on Figure 3. The microstructure of the films consists of a number of grains, which are uniformly distributed throughout the surface. The surface morphology of the 1 mol% Mo-doped ZnO thin film shows small dense grains. When the concentration increases to 2%, the number of nuclei increases, and the nuclei grow over the entirety of the surface area of the substrate with uniform grains. The grain size of the pure ZnO thin films is approximately 30 nm. The images reveal that the grain size of the Mo-doped films increases to 60–80 nm compared to the pure ZnO thin films. This could be explained as a result of the migration ability of the atoms on the surface. Additionally, the morphology of the ZnO films depends on many factors, such as: the flow of the solution, substrate temperature, carrier gas, precursors, oxidant source, distance between nozzle and spray, etc. [18]. When there is a fairly high flow of the carrier gas during the solid-phase reaction, the grains’ sizes increase due to their high capacity for building aggregates. The authors A. Zak et al. identified a decrease in the grain size, which might be closely related to the lower flow of carrier gas [24].

**Figure 4:**
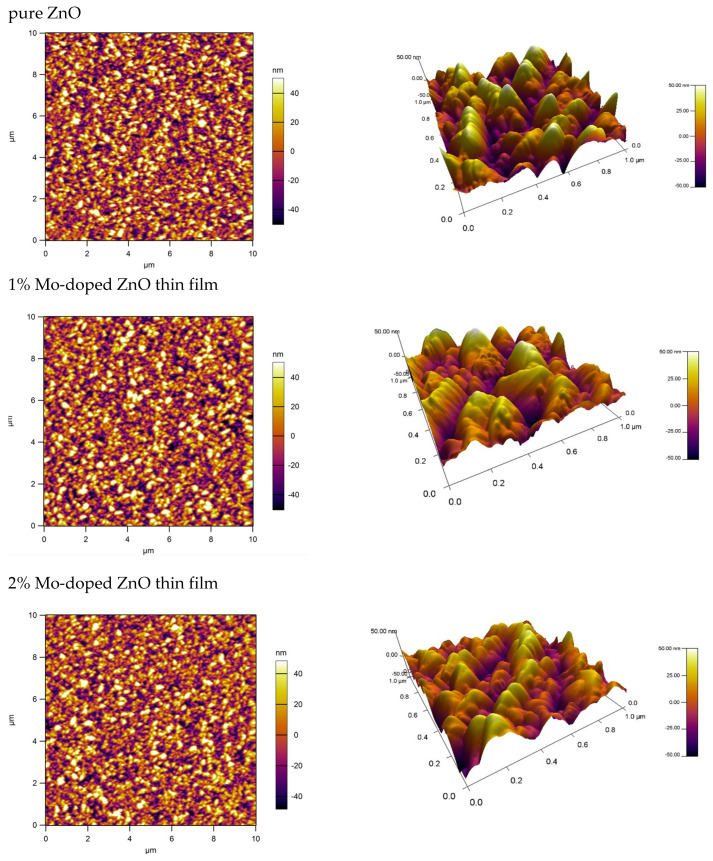
AFM 2D and 3D images of the pure and 1% and 2% Mo-doped ZnO thin films.

Figure 4 shows AFM 2D and 3D images of pure ZnO and doped ZnO thin films. The scanning areas are 20 µm × 20 µm and 1 µm × 1 µm. The films have uniform granular structures and the grains are of nanometer size. The particle size of the Mo-ZnO films is found to increase with the increase in the concentration of molybdenum, which corresponds to the SEM results. The average size of the grains is between 60 and 80 nm, calculated for a 1 µm × 1 μm film.

The evaluated root mean square (RMS) roughnesses of the undoped and 1% and 2% Mo-doped ZnO thin films, assessed by atomic force microscopy (AFM), are 6.12, 23.54, and 23.83 nm, respectively. This study shows that the surface roughness is higher for Mo-doped thin films compared to pure films. 

## 4. Conclusions

The effects of Mo on the structure, optical properties and morphology of ZnO thin films prepared on glass and silicon substrates were investigated. XRD patterns indicate the presence of polycrystalline films with a wurtzite structure. The intensity of the diffraction peaks alternates, indicating modifications in the structures of doped films. SEM analysis showed that the microstructure of the films consists of dense grains uniformly distributed throughout the surface. The AFM studies reveal that the film’ surface roughness increases when adding Mo as a dopant, and with the concentration of Mo as well. UV-VIS spectroscopy data show an increase in transmittance for Mo-doped ZnO thin films in the visible region. 

## Figures and Tables

**Table 1:** The influence of the Mo content on the optical properties.

	*Refractive Index, n*	*Extinction Coefficient, k*	λ1, *nm*	λ2, *nm*	*Calculated Film Thickness* *, nm*	*Measured Film Thickness, nm*	*Eg, eV*
*pure ZnO*	*1.76*	*0.007*	*345*	*470*	*324*	*350*	*3.341*
*1% MoO_3_*	*1.885*	*0.017*	*330*	*480*	*264*	*244*	*3.279*
*2% MoO_3_*	*1.792*	*0.025*	*345*	*500*	*278*	*260*	*3.291*

## Data Availability

The original contributions presented in the study are included in the article, further inquiries can be directed to the corresponding author.

## References

[1] Swapna R., Santhosh Kumar M.C. (2012). The role of substrate temperature on the properties of nanocrystalline Mo doped ZnO thin films by spray pyrolysis. Ceram. Int..

[2] Fayaz Rouhi H., Rozati S.M. (2022). Synthesis and investigating effect of tellurium-doping on physical properties of zinc oxide thin films by spray pyrolysis. Appl. Phys. A.

[3] KaniAmuthan B., Vinoth S., Karthikeyan V., Roy V.A.L., Thilakan P. (2019). Influence of nitrogen dopant source on the structural, photoluminescence and electrical properties of ZnO thin films deposited by pulsed spray pyrolysis. Ceram. Int..

[4] Ganesh V., Anila K., Jayarama A., Bhat S., Rai C.S., Pinto R. (2022). Spray pyrolysis deposited aluminium-indium zinc oxide thin films and study their electrical and photoluminescence properties. Mater. Today Proc..

[5] Chen S., Carraro G., Barreca D., Binions R. (2015). Growth and electro-optical properties of Ga-doped ZnO films prepared by aerosol assisted chemical vapour deposition. Thin Solid Films.

[6] Vettumperumal R., Kalyanaraman S., Thangavel R. (2015). Effect of Er concentration on surface and optical properties of K doped ZnO sol-gel thin films. Superlattices Microstruct..

[7] Hegazy A.R., Salameh B., Alsmadi A. (2019). Optical transitions and photoluminescence of fluorine-doped zinc tin oxide thin films prepared by ultrasonic spray pyrolysis. Ceram. Int..

[8] Makino H., Shimizu H. (2018). Influence of crystallographic polarity on the opto-electrical properties of polycrystalline ZnO thin films deposited by magnetron sputtering. Appl. Surf. Sci..

[9] MKhan M., Bhatti K., Qindeel R., Alonizan N., Althobaiti H.S. (2017). Characterizations of multilayer ZnO thin films deposited by sol-gel spin coating technique. Results Phys..

[10] Raphael R., Anila E. (2021). Investigation of photoluminescence emission from β-Ga_2_O_3_:Ce thin films deposited by spray pyrolysis technique. J. Alloys Compd..

[11] Oh B.-Y., Jeong M.-C., Lee W., Myoung J.-M. (2005). Properties of transparent conductive ZnO:Al films prepared by co-sputtering. J. Cryst. Growth.

[12] Purica M., Budianu E., Rusu E. (2001). ZnO thin films on semiconductor substrate for large area photodetector applications. Thin Solid Films.

[13] Shewale P., Sim K.U., Kim Y.-B., Kim J.H., Moholkar A.V., Uplane M.D. (2013). Structural and photoluminescence characterization of SnO_2_: F thin films deposited by advances spray pyrolysis technique at low substrate temperature. J. Lumin..

[14] Gupta S., Yadav B., Dwivedi P.K., Das B. (2013). Microstructural, optical and electrical investigation of Sb-SnO_2_ thin films deposited by spray pyrolysis. Mater. Res. Bull..

[15] Shirpay A., Mohagheghi M.B. (2022). Dependence of structure and energy band gap on sensing properties of WO_3_:TeO_2_ thin films deposited by spray pyrolysis. Phys. B.

[16] Chen G., Zhao X., Zhang H., Liu F., Wang Y., Wang H., Gao J., Zhao Y., Li W., Tao J. (2016). Effect of growth rate on the structure and physical properties of Mo doped ZnO films. Superlattices Microstruct..

[17] Swapna R., Kumar M.S. (2013). Growth and characterization of molybdenum doped ZnO thin films by spray pyrolysis. J. Phys. Chem. Solids.

[18] Zhao D., Sathasivam S., Li J., Carmalt C.J. (2020). Transparent and conductive Molybdenum-Doped ZnO thin films via chemical vapor deposition. Appl. Electron. Mater..

[19] Berberova-Buhova N., Nedelchev L., Stoykova E., Nazarova D. (2022). Optical response evaluation of azopolymerthin solid films doped with gold nanoparticles with different sizes. J. Chem. Technol. Metall..

[20] Berberova N., Sharlandjiev P., Stoilova A., Nedelchev L., Nazarova D., Blagoeva B. (2018). Optical constants of azopolymer PAZO thin films in the spectral range 320–800 nm. J. Phys. Conf. Ser..

[21] Prasada Rao T., Santhoshkumar M.C. (2009). Highly oriented (100) ZnO thin films by spray pyrolysis. Appl. Surf. Sci..

[22] Jiles D. (1994). Introduction to the Electronic Properties of Materials.

[23] Bhuvana K.P., Elanchezhiyan J., Gopalakrishnan N., Balasubramanian T. (2011). Influence of grain size on the properties of AlN doped ZnO thin film. Mater. Sci. Semicond. Process..

[24] Zak A.K., Ghanbari A., ShekoftehNarm T. (2017). The effect of molybdenum on optical properties of ZnO nanoparticles in Ultraviolet-Visible region. Adv. Powder Technol..

